# Digital adherence technologies for the management of tuberculosis therapy: mapping the landscape and research priorities

**DOI:** 10.1136/bmjgh-2018-001018

**Published:** 2018-10-11

**Authors:** Ramnath Subbaraman, Laura de Mondesert, Angella Musiimenta, Madhukar Pai, Kenneth H Mayer, Beena E Thomas, Jessica Haberer

**Affiliations:** 1 Department of Public Health and Community Medicine and Tufts Center for Global Public Health, Tufts University School of Medicine, Boston, Massachusetts, USA; 2 Division of Geographic Medicine and Infectious Diseases, Tufts Medical Center, Boston, Massachusetts, USA; 3 Division of Infectious Diseases, Brigham and Women’s Hospital, Boston, Massachusetts, USA; 4 Department of Information Technology, Mbarara University of Science and Technology, Mbarara, Uganda; 5 McGill International TB Centre, McGill University, Montreal, Quebec, Canada; 6 The Fenway Institute, Fenway Health, Boston, Massachusetts, USA; 7 Division of Infectious Diseases, Beth Israel Deaconess Medical Center, Boston, Massachusetts, USA; 8 Department of Social and Behavioural Research, National Institute for Research in Tuberculosis, Chennai, Tamil Nadu, India; 9 Center for Global Health, Massachusetts General Hospital, Boston, Massachusetts, USA

**Keywords:** tuberculosis, medication adherence, differentiated care, SMS reminders, mhealth, mobile technologies, electronic medication packaging devices, digital medication monitors

## Abstract

Poor medication adherence may increase rates of loss to follow-up, disease relapse and drug resistance for individuals with active tuberculosis (TB). While TB programmes have historically used directly observed therapy (DOT) to address adherence, concerns have been raised about the patient burden, ethical limitations, effectiveness in improving treatment outcomes and long-term feasibility of DOT for health systems. Digital adherence technologies (DATs)—which include feature phone–based and smartphone-based technologies, digital pillboxes and ingestible sensors—may facilitate more patient-centric approaches for monitoring adherence, though available data are limited. Depending on the specific technology, DATs may help to remind patients to take their medications, facilitate digital observation of pill-taking, compile dosing histories and triage patients based on their level of adherence, which can facilitate provision of individualised care by TB programmes to patients with varied levels of risk. Research is needed to understand whether DATs are acceptable to patients and healthcare providers, accurate for measuring adherence, effective in improving treatment outcomes and impactful in improving health system efficiency. In this article, we describe the landscape of DATs that are being used in research or clinical practice by TB programmes and highlight priorities for research.

**Summary box**

- Digital adherence technologies (DATs)—which include feature phone–based and smartphone-based technologies, digital pillboxes and ingestible sensors—have the potential to facilitate more patient-centric approaches for monitoring tuberculosis (TB) medication adherence than existing directly observed therapy (DOT) models.
- DATs may serve a variety of functions in TB care, including reminding patients to take their medications, facilitating digital observation of pill-taking, compiling patient dosing histories and triaging patients based on their level of adherence, which can facilitate provision of individualised (‘differentiated’) care.
- Evidence that DATs improve TB treatment outcomes is limited, and more robust research is needed to understand the acceptability, accuracy, clinical effectiveness and cost-effectiveness of these technologies.
- DATs should be integrated with clinical strategies for identifying and addressing the underlying psychosocial, medical, structural and health system–related causes of medication non-adherence; otherwise, implementation of DATs may run the risk of overly focusing on ‘observation’ and replicating paternalistic aspects of existing DOT models.

## Introduction

Tuberculosis (TB) is the leading infectious cause of death globally, even though most forms of TB are curable.^1^ The risks of death, disease relapse and acquired drug resistance increase with irregular adherence to TB therapy.^2 3^ Compared with drug-sensitive TB, drug-resistant strains require an extended duration of therapy with second-line or third-line drugs that are less effective, have higher rates of adverse effects and are more expensive.

Causes of medication non-adherence are complex and include psychosocial (eg, alcohol use,^4^ depression,^5^ stigma^6^), structural (eg, distance from clinics, medication costs), therapy-related (eg, toxicities^5^) and health system–related barriers (eg, lack of counselling, poor user-experience with the health system).^7 8^ Directly observed therapy (DOT) was designed to reduce non-adherence; however, concerns have been raised that some DOT approaches may impinge on patient autonomy^9^ and have minimal efficacy for improving treatment outcomes, as compared with self-administered therapy.^10–12^

With the expansion of mobile phone and cellular access—including in high-TB-burden countries in Africa,^13^ Asia and Latin America—digital adherence technologies (DATs) may facilitate alternative approaches for improving adherence. These technologies range from cellphone short messaging service (SMS) texts, to digital pillboxes, to ingestible sensors. DATs use cellular communication and other innovations to perform a variety of functions, including reminding patients to take medications, digitally observing doses taken and compiling dosing histories that can be used by healthcare providers (HCPs) to identify and intervene on non-adherence. DATs have been shown to improve adherence in patients with HIV,^14–17^ diabetes^18^ and other conditions.^19 20^ Fewer studies have evaluated whether these changes in adherence translate into better clinical outcomes, such as a recent study that found improved viral suppression in patients with HIV enrolled in a DAT-based adherence intervention.^21^

DATs may be particularly relevant for rethinking TB care delivery, for a few reasons. First, improving TB medication adherence may have public health benefits, such as reduced rates of disease relapse, acquired drug resistance and transmission of infection.^2 3^ Second, unlike other diseases for which self-administered therapy is the standard of care, many TB programmes globally currently use DOT for monitoring.^22^ While some TB programmes may view DATs as a challenge to their existing DOT models, in many contexts, DATs may provide an alternative for ‘observing’ medication adherence, potentially making them more acceptable to patients with TB and HCPs than they are for other diseases, as is discussed further below. Finally, unlike chronic diseases that require lifelong treatment (eg, hypertension), TB treatment has a defined duration, such that monitoring the entire treatment course with DATs may be feasible.

DATs are being deployed for TB care in research and routine clinical practice in several countries, such as China, India and Belarus, prompting publication of a handbook by WHO regarding their use.^23^ A recent systematic review summarised the evidence on whether use of DATs improves TB medication adherence and treatment outcomes.^24^ This review found that SMS-based strategies have not been found to improve treatment completion rates in settings with suboptimal outcomes at baseline. The review found similarly high rates of treatment completion when comparing treatment monitoring by video DOT and in-person DOT in high-income country settings. In addition, the review found two studies suggesting that digital pillboxes may reduce missed doses and increase the probability of cure in some contexts. On the whole, however, that systematic review found the evidence supporting use of DATs for TB to be limited, and the authors suggest that more robust evidence is needed to understand how these technologies may impact patients and health systems.^24^

While that previous systematic review described the existing evidence on use of DATs for TB, this current narrative review has a different goal. We use the findings of that prior systematic review as a starting point for describing the landscape of existing technologies that are currently being used and for highlighting critical gaps in research. Our review has been guided by a systematic search of the literature from 2000 to mid-2017 to ensure we cover the breadth of DATs currently being used in TB care (box 1). We first provide historical context for the use of DATs for TB and describe a conceptual framework that can inform their integration into clinical care. We then describe the variety of DATs that are being used for monitoring TB medication adherence and discuss key functions of DATs that have the potential to benefit patients and health systems. Finally, we highlight research priorities that could help to refine the testing and evaluation of DATs and create an evidence base to better understand their benefits and limitations for TB care.

Box 1 **Search strategy and selection criteria**

To better inform our narrative review, we searched PubMed for peer-reviewed articles published between 1 January 2000 and 31 July 2017, with the terms referring to tuberculosis (eg, ‘tuberculosis’ OR ‘TB’ OR ‘Mycobacterium tuberculosis’) and terms that refer to digital adherence technologies generally (eg, ‘adherence technology’ OR ‘mHealth’ OR ‘mobile technology’ OR ‘digital medication monitors’ OR ‘electronic monitors’ OR ‘remote observation’) as well as specific technologies (eg, ‘short messaging service’ or ‘cellphone’ or ‘smartphone’ or ‘digital pillboxes’ OR ‘electronic medication packaging’ OR ‘video DOT’ OR ‘ingestible sensors’). We only started our search after the year 2000 since these technologies are relatively new in the last 15 years. We selected case reports, qualitative studies, cohort studies, randomised trials and systematic reviews published in English. We also reviewed the references sections of these articles and sought advice from experts in the field to identify additional studies. We did not exclude any studies based on the methodology used or the study quality. We specifically excluded articles focused on technologies used to support medication adherence during treatement for latent TB infection.

## Rethinking the DOT model

The idea of direct observation of medication ingestion for TB emerged in the 1950s and 1960s from studies in Hong Kong and India.^25^ Some of the perceived strengths of direct observation included close monitoring of adherence, face-to-face interactions between patients and HCPs, and careful documentation of treatment records. In 1991, the World Health Assembly adopted the ‘directly observed therapy, short-course’ (DOTS) strategy.^22^ DOTS is a multipronged intervention for which direct observation of therapy is just one component. DOTS also included use of ‘short-course’ therapy (ie, 6 months), use of smear microscopy for diagnosis and systematic reporting of treatment outcomes.^22^ While treatment success rates globally improved under DOTS,^22^ the extent to which these improvements can be attributed to direct observation of therapy versus other aspects of the DOTS package are unclear.

Recent systematic reviews suggest that DOT does not achieve superior results compared with self-administered therapy (SAT) across a variety of outcomes—including treatment completion,^10 12^ microbiological cure,^10 12^ microbiological failure,^11^ disease relapse^11^ and acquired drug resistance.^11^ Interviews with patients with TB show that DOT may be associated with perceptions of low autonomy, inadequate confidentiality and stigma.^9 26 27^ In settings using facility-based DOT, frequent health facility visits may result in loss of income and employment.^9 26 28^

DOT also raises challenges for health systems. Most DOT models assume that all patients require uniform monitoring—placing a high burden of supervision on HCPs—rather than stratifying patients by their level of non-adherence, so that HCPs can focus resources on the highest-risk patients. As a result of these challenges, in practice, DOT is poorly implemented, or not strictly adhered to in practice, in many TB programmes, especially where community DOT is used.^27 29–31^ In light of the limitations of DOT, DATs have the potential to provide more patient-centric approaches for ‘observing’ pill-taking,^32^ to reduce financial burdens incurred by patients from frequent health facility visits and to identify non-adherent patients so HCPs can better focus their efforts.^33^

## Landscape and functions of TB adherence technologies

**Figure 1:**
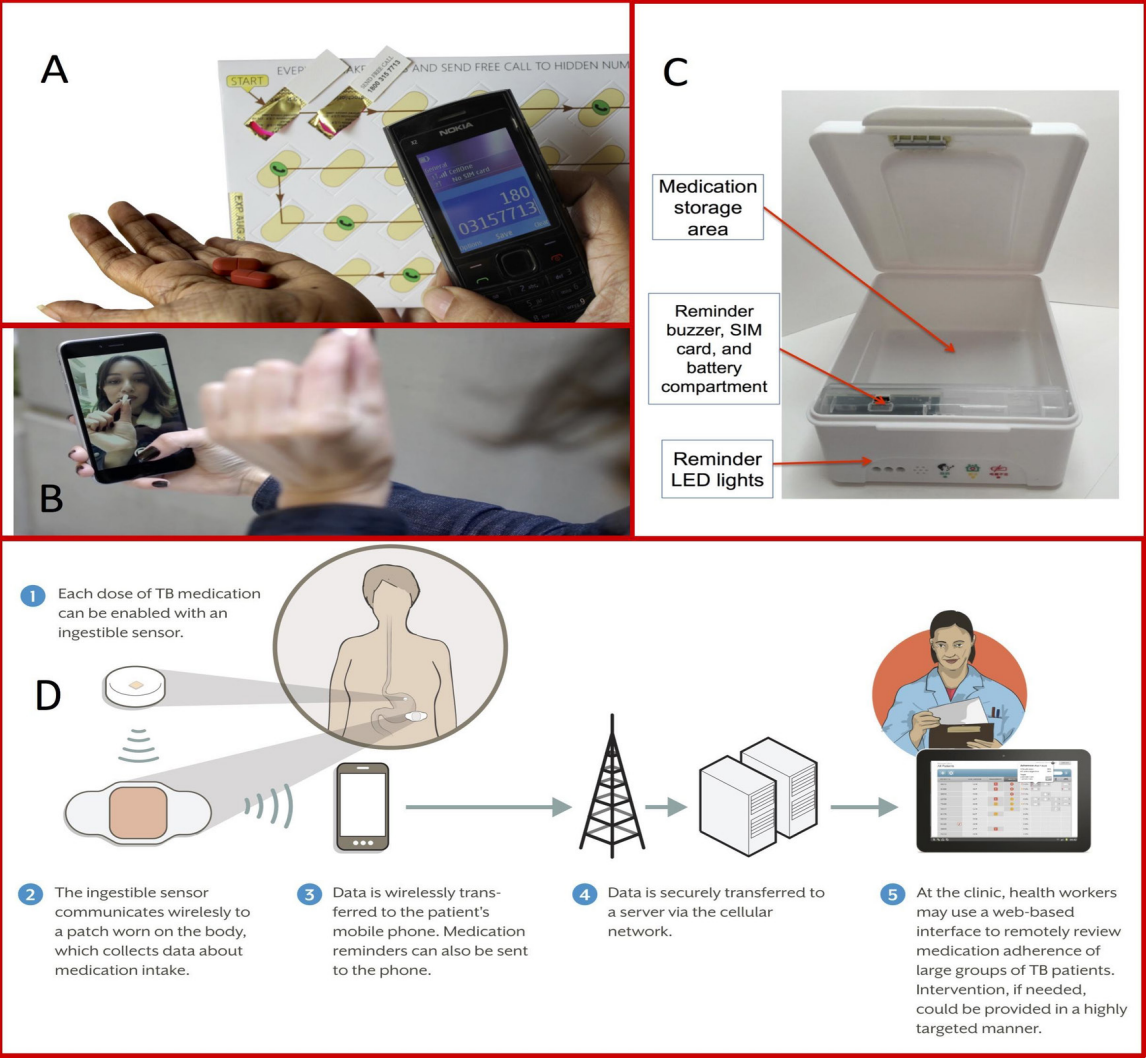
Examples of different adherence monitoring technologies. (A) 99DOTS, a feature phone-based adherence technology (with permission from Everwell Health Solutions);^87^ (B) SureAdherence, a video DOT strategy (with permission from SureAdherence Mobile Technologies);^53^ (C) evriMED, a digital pillbox (with permission from the Wisepill Technologies);^88^ (D) an ingestible sensor–based adherence monitoring approach (Source: Belknap et al.^37^). DOT, directly observed therapy; LED, light-emitting diode; SIM, subscriber identification module; TB, tuberculosis.

**Figure 2:**
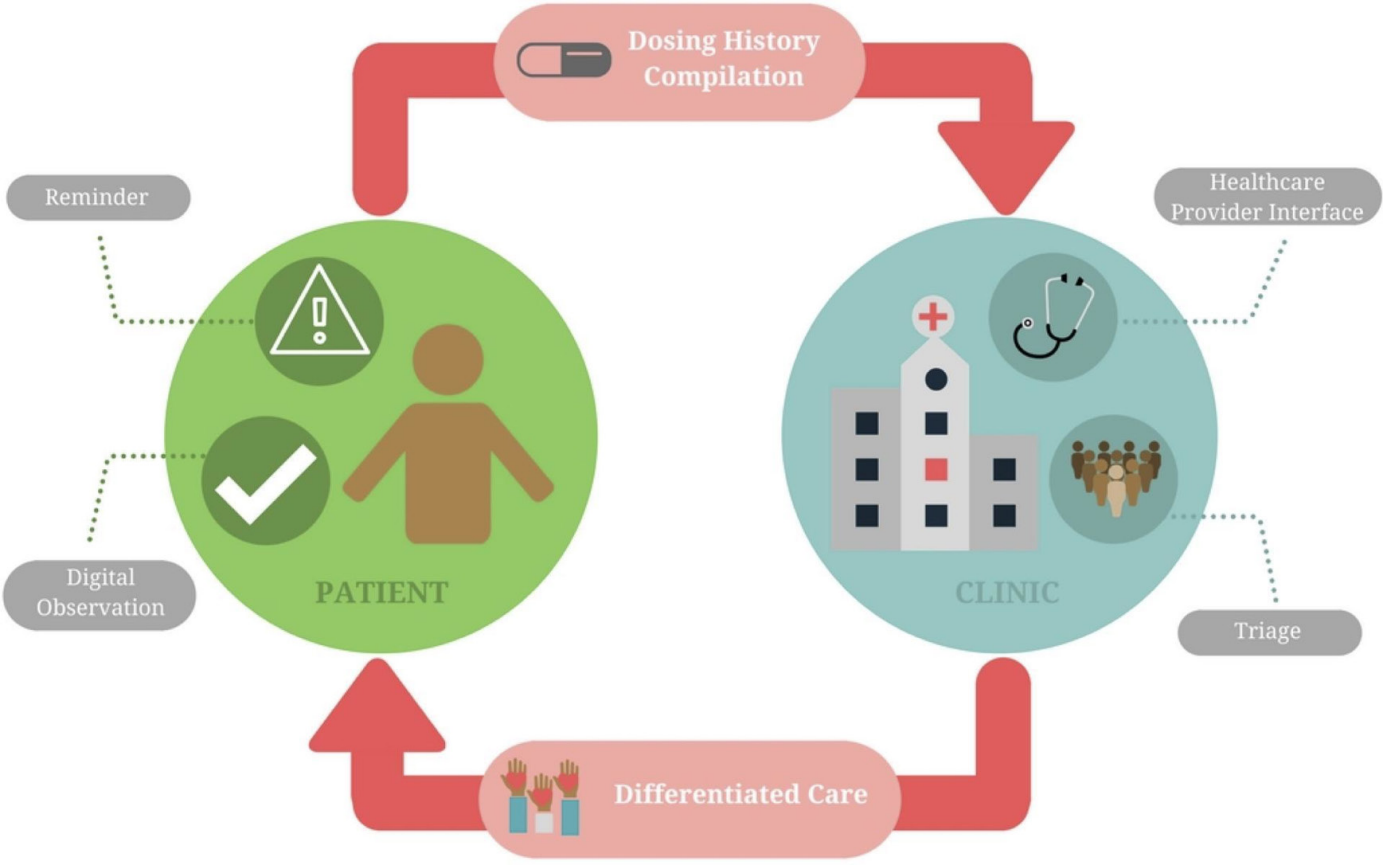
Functions that digital adherence technologies (DATs) can play to reinforce patient medication adherence and facilitate monitoring and triage of patients by health systems. ‘Differentiated care’ refers to providing different intensities and types of care based on a patient’s level of medication adherence as measured by the DAT.

We describe select DATs that are being used in research or clinical care for TB in table 1 and figure 1, along with details regarding SAT and DOT approaches for comparison. In SMS-based strategies, SMS texts remind patients to take medications; many approaches allow the patient to send a SMS response (ie, ‘two-way’) to indicate the dose has been taken. With 99DOTS, patients are issued TB medications in blister packs wrapped in a custom envelope. When a dose is dispensed, a hidden phone number is revealed on the inner envelope flap, prompting the patient to place a toll-free call to indicate a dose taken.^34^ In video DOT (VDOT), video conferencing via smartphone or computer allows HCPs to watch patients take medications, either synchronously (in real time) or asynchronously (at a different time using recorded video). Digital pillboxes have pre-programmed audiovisual reminders embedded in the pillbox. Opening and closing the box to access medications is recorded as a proxy for a dose taken. Ingestible sensors are microchips embedded in TB medications. Contact with a patient’s gastric fluid after ingestion results in transmission of a signal to an adhesive monitor worn by the patient. From there, the information is transmitted to the patient’s smartphone and then to a server, where HCPs can access dosing histories. Online supplementary appendix 1 provides more extensive details on each DAT. Below, we describe the key functions that DATs may perform in patient care (figure 2).

10.1136/bmjgh-2018-001018.supp1 Supplementary data

**Table 1:** Summary of medication adherence monitoring strategies and technologies currently being pilot-tested or implemented in clinical settings for tuberculosis care

	Description of monitoring approach or technology	Estimated range of costs in US dollars (select examples of technologies)	Sites of implementation	Reminder function	Approach to digital observation	Healthcare provider (HCP) interface	Triage function
Self-administered therapy (SAT)	Patients take medications themselves without any formal dose observation strategy.^10^ Clinic visits may be combined with additional adherence monitoring approaches, such as urine isoniazid testing^71^; however, this has not been done routinely in national TB programmes.	Variable based on the setting. This represents the base cost of care provision, with most adherence monitoring strategies outlined below adding costs on top of this value.	Standard of care in countries not implementing DOT. De facto standard of care in settings where DOT is not functioning optimally.^27 29–31^	Reminders about adherence may take place during routine clinic visits.	Adherence evaluation may take place during clinic visits via basic questions asked by HCPs to patients or less commonly by pill counts.^59^	Face-to-face interactions during follow-up visits.	Patients are generally provided with uniform (undifferentiated) care, though referrals to counsellors and other services are possible.
Directly observed therapy (DOT)	Facility-based DOT: patient visits health facility to be observed taking every medication dose (most common DOT model in LMICs).^10^ Home-based DOT: HCP visits a patient’s home to observe her take each dose.^10^ Community-based or family-based DOT: family member or community resident observes patient taking each dose.^10^	Variable based on the setting and DOT model, with facility-based models generally having lower costs than in-person DOT using home visits, due to lower personnel and travel costs.	Standard of care for monitoring TB medication adherence in many countries.^22 89^	Reminders are not routine; however, the health system is supposed to take prompt action if patients do not show up to facilities for DOT.	HCP or other designated individual observes a patient swallow the dose.	Frequent face-to-face interactions with HCPs or other designated individual.	Patients are generally provided with uniform care, regardless of the risk of non-adherence.
Short message service (SMS)–based strategies	SMS texting can remind patients to take TB medication doses (one-way SMS).^39 68 79^ Patients may respond by SMS text or phone call to indicate a dose taken (two-way SMS). Often used in combination with other DATs.^39 90^	In nearly all settings, costs are generally low (eg, less than US$1 to US$2 per patient per treatment course), assuming that patients can access a feature phone.	Interventions in numerous African countries,^78 79 91–94^ China,^39^ ^95^ Indonesia,^96^ Thailand,^97^ India^90^ and Pakistan^68^ have used one-way SMS reminders, or two-way SMS alone or in combination with other adherence monitoring strategies.^98^	Prescheduled, automated SMS text reminders can be sent to a patient’s mobile phone each day and repeated multiple times (or reminders sent to HCPs or family members) if patients do not respond to report a dose taken.^68 78 79^	Patients respond to the reminder SMS via response SMS text or free call.^68 78 79^	HCPs access dosing histories compiled from patients’ SMS or phone call responses through online portals accessible on computers or smartphones.^79 90^	Patients who do not respond to reminder SMS texts can be triaged to receive additional reminder texts or personalised SMS texts or phone calls from HCPs encouraging them to continue therapy or return to the clinic for evaluation.^68 79^
99DOTS	TB medications are issued in blister packs wrapped in an envelope. On dispensing a dose, a hidden phone number is revealed on the inner envelope flap, prompting the patient to place a toll-free call to indicate a dose taken.^34 87^	Estimated cost per patient per treatment course in LMIC settings is US$5 to US$6, with roughly half of costs related to the custom envelopes and half related to technology, including communication costs for SMS texts and missed calls. This assumes patients can access a feature phone for calling the toll-free numbers.^34^	Over 150,000 patients with TB have been registered in India, along with a smaller number in Myanmar.^34^	Patients receive automated SMS reminders every day, with additional reminders if doses are missed.	Phone numbers that are unpredictable to the patient are revealed with each dispensed dose. Calling the phone number therefore indicates that a specific dose was taken.^34 87^	HCPs can receive SMS text notifications regarding potentially non-adherent patients and monitor patients’ adherence in real time through an online portal accessible on computers or smartphones.^34 87^	Patients are triaged into risk groups based on the frequency of unreported doses. HCPs can follow up with phone calls or home visits.^34^
Video DOT (VDOT)	Synchronous VDOT: prescheduled live-streaming video conferencing through a secure interface allows an HCP to watch a patient take her TB medications at home in real time.^67^ Asynchronous VDOT: patient sends a pre-recorded video of herself taking medications using a smartphone or webcam to HCP, who views the video and confirms adherence.^53 64^ ‘Observation’ can also be automated by use of facial recognition and medication identification software, saving time for HCPs.^35^	For a 6-month course of daily treatment, subscription costs for the SureAdhere application are approximately US$210 (US$35 per month) in developed countries and US$24 (US$4 per month) in LMICs. For patients who do not already have a smartphone or tablet with data services, the estimated additional cost for a TB programme in the USA to equip their patients is approximately US$324 (US$54 per month) for data services and US$100 for a smartphone. Data services may be less expensive in LMICs than in high-income countries.	Mostly middle-income and high-income countries (eg, Mexico, USA,^99^ England, Singapore) where smartphones are reliable and widely available,^35 53 64 67 100^ though pilot studies have been conducted in Kenya^54^ and Vietnam.^101^	SMS texts can be sent to remind patients of their next videoconferencing appointment or to record and submit a video.^53 67^	Patient names and swallows each pill in front of the camera.^67^ HCP observes the dose live or asynchronously,^53 67^ or ‘observation’ can be automated using facial recognition and medication identification software.^35^	Live-streaming VDOT interface has benefits other than observation since HCPs can ask patients about medication adverse effects.^67^ Computer portal also shows patient’s dosing history.	Uniform (undifferentiated) care is provided to patients. Missed VDOT appointments or pre-recorded videos are followed up by phone calls or home visits.^53 67^
Digital pillboxes	Digital pillboxes store TB medications and have pre-programmed audiovisual reminders embedded in the pillbox. Opening and closing the box to access medications serves as a proxy indicator of a dose taken. This information is transmitted via a SIM card to create a real-time dosing history accessible by HCPs.^39 57 102^	Device costs range from as low as US$14 for evriMED cardboard frame devices that do not provide data in real time to US$23 for evriMED plastic frame devices that deliver information in real time, to US$130 for Wisepill devices that provide information in real time.	Used in research studies for patients with drug-susceptible (DS) TB in China,^39^ ^57^ Tanzania^103^ and Uganda and for patients with DS and MDR TB in India.^88^	Present as digital displays, alarms or automated voice alerts integrated within the pillbox. Patient stops receiving reminders for the day after the box has been opened.^39^	Opening the digital pillbox serves as a proxy indicator for a dose taken, though it does not ensure actual ingestion. Failure to open the pillbox on a given day serves as a proxy indicator for a missed dose.	HCPs can view dosing histories through an online portal or get alerts about missed patient doses via SMS.^39 102^	In a study in China, patients who missed 3 to 6 doses based on a digital pillbox–compiled history were triaged to a weekly HCP visit and patients who missed seven or more doses were triaged to in-person DOT.^39^
Ingestible sensors	Ingestible sensors are microchips embedded in TB medications. After the dose is ingested, the ingestible sensor interacts with a patient’s gastric fluid and transmits a signal to an adhesive monitor worn by the patient. The monitor transmits pill-taking information to the patient’s smartphone, which transmits information to a server to allow HCPs to access dosing histories.^37 38^	Costs not currently available.	Used in pilot studies in the USA.^37 38^	Current ingestible sensor models do not have a reminder function; however, reminders can be sent to patients’ smartphones.^38^	Ingestible sensors confirm actual medication ingestion since signal transmission happens when the ingestible sensor contacts gastric juices; however, patients must consistently wear the adhesive patch and have smartphone access.^37 38^	HCPs use an online portal to access dosing histories compiled by the adhesive monitor and transmitted via smartphone to a server.^37 38^	Triage strategies not defined in previous studies, but dosing histories may allow providers to identify non-adherent patients and provide differentiated care.^38^

DAT, digital adherence technology; DS TB, drug-susceptible tuberculosis; HCP, healthcare provider; LMIC, low-income and middle-income country; MDR TB, multidrug-resistant TB; SIM, subscriber identification model.

### Reminder function

DATs provide reminders to patients, addressing forgetfulness, which is a common barrier to adherence.^8^ Forgetfulness is commonly thought of as a cognitive barrier to adherence, but it also reflects psychosocial and structural barriers faced by patients, such as forgetting doses due to alcohol use or working multiple jobs. Reminders may promote habit formation in pill-taking behaviour.^15 32^ For most DATs, reminders take the form of SMS texts. Digital pillboxes have embedded audiovisual reminders (eg, glowing light and ringing sound), which have the benefit of prompting patients to the site where medications are stored.

### Digital observation of dose-taking

Most DATs digitally ‘observe’ or record dose-taking, which is especially relevant in TB given the historical reliance on DOT. VDOT mimics DOT by allowing HCPs to view patients swallow pills. As with DOT, VDOT may raise concerns about patient autonomy—as patients may feel that being watched taking every dose is an invasion of privacy—although new technologies using automated facial recognition and pill identification could obviate the need for HCPs to watch every video.^35 36^ For some DATs, such as two-way SMS-based strategies, 99DOTS and VDOT, an extra step is needed to report dose-taking that requires effort by the patient herself, such as responding to an SMS text, placing a phone call or getting on a video call. For these technologies, dosing histories are compiled based on patient responses, and inaccuracies in the dosing history may be introduced because patients could send SMS responses or phone calls without taking doses (ie, over-reporting) or take doses without sending SMS responses or phone calls (ie, under-reporting).

Digital pillboxes may minimise patient effort and the risk of HCPs accidentally mis-recording information on paper records because opening and closing the pillbox is digitally recorded as a ‘dose taken’. However, there are other potential limitations to their accuracy—for example, if a patient removes medication blister packs from the pillbox, allowing doses to be taken without opening it. Ingestible sensors record pill-taking with relatively high sensitivity and specificity^37 38^; however, removal of the adhesive monitor that records information transmitted by the ingested sensors would result in under-reporting of adherence. For all of these strategies, inaccuracy in patient reporting may be reduced by patient education about the purpose and appropriate use of these devices.

### Compilation of dosing histories

**Figure 3:**
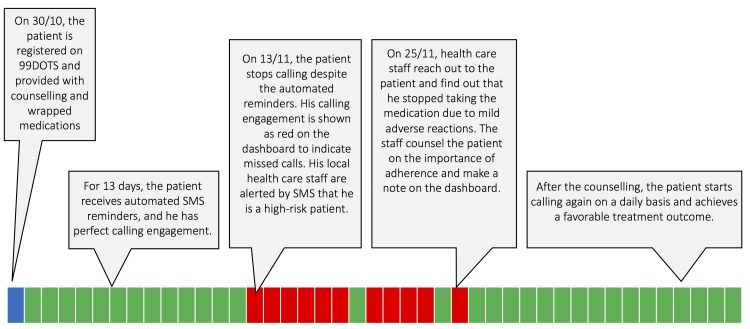
Example of how digital adherence technologies involving daily reporting of dose-taking could potentially facilitate earlier identification and intervention to address medication non-adherence. The 99DOTS model is used for illustrative purposes. Each box represents a calendar day on the dashboard viewed by healthcare providers. Green boxes represent doses that were ‘called in’ on a given day and red boxes represent doses that were not called in. SMS, short messaging service.

DATs compile patient dosing histories, which allow for ‘real-time’ or clinic visit–based adherence monitoring. In real-time approaches, doses are recorded right after the patient engages the technology (eg, opening the pillbox or sending an SMS response). HCPs remotely access these histories on a web-based interface, allowing them to identify non-adherence before the patient’s next medication refill visit (figure 3). In clinic visit–based monitoring, HCPs access dosing histories during patient visits, for example by uploading the record to a computer from the patient’s digital pillbox. HCPs can then counsel patients using dosing information compiled since the last clinic visit. For example, in a study from China, at each clinic visit, the doctor reviewed the patient’s dosing history for the prior month on a computer, discussed the reason for missed doses and switched patients who missed numerous doses to more intensive management strategies.^39^

### Triage and provision of differentiated care

In contrast to DOT, in which patients are treated using a uniform approach, DATs can facilitate triage of patients based on different levels of adherence. Triage may be performed by HCPs during routine reviews of dosing histories. Alternatively, computer algorithms can be used to triage and alert HCPs about non-adherent patients to prompt early intervention, saving time for HCPs and potentially preventing patient loss to follow-up (figure 3). Triage can then facilitate escalation in the intensity of care for patients with a high level of non-adherence. For example, in a randomised trial of DATs in China, patients who missed three to six medication doses were switched to ‘intensive management’ consisting of weekly HCP visits to the patient’s home. Those who missed >7 doses were switched to DOT.^39^

While DATs may help identify poor adherence, a careful clinical evaluation is needed to address its causes, which are often complex and may include psychosocial, treatment-related and health system–related barriers. As such, identification of non-adherence using DATs should ideally be viewed as the starting point for more intensive face-to-face engagement with patients to understand their specific reasons for non-adherence, so that individualised packages of care can be provided (ie, ‘differentiated care’). By triaging patients, HCPs may be able to spend more time on these high-risk patients to address their needs. Triage and differentiated care have the potential to focus limited resources on higher-risk patients, which may improve the efficiency of care delivery.

## Research priorities and existing evidence

### Acceptability and ethical questions

Acceptability may differ based on the type of DAT and the context in which it is deployed. Development of DATs should ideally follow a participatory approach that iteratively incorporates views of users and stakeholders—including patients with TB, caregivers and HCPs—in order to improve acceptability.^40 41^ Models that have been shown to predict acceptance of health technologies, such as the Unified Theory of Acceptance and Use of Technology and the Technology Acceptance Model, can guide evaluations of DAT acceptance.^42–44^ In general, these models evaluate ‘ease of use’ (ie, how easily users are able to learn the DAT), ‘perceived usefulness’ (ie, whether users think it is valuable for TB care) and the availability of infrastructure to support their use. Frameworks have also been proposed for evaluating ethical aspects of DATs.^45 46^ These frameworks emphasise evaluation of patient autonomy (including concerns about ‘surveillance’),^47^ privacy and confidentiality, stigma and intrusiveness, and trust between patients and HCPs.^45 46^

Patient literacy—with regard to language (for two-way SMS), using a feature phone (for two-way SMS or 99DOTS) or using a smartphone (for VDOT or ingestible sensors)—should be assessed in any evaluation of acceptability. In populations with low language literacy, providing reminders in local languages or voice messages (rather than SMS texts) may expand the reach of some DATs. Researchers should also assess optimal programming of a DAT’s reminder function. Reminders that are programmed for the wrong times—for example when patients are sleeping or at work—may make it difficult to respond in a timely manner. Overly frequent reminders may result in patients opening pillboxes just to shut off reminders or discarding SMS texts before they are read.^39^

Maintaining privacy of a patient’s TB diagnosis is an important aspect of acceptability^45 46^ because stigma can result in discrimination or negatively affect a patient’s coping capacity.^6 48^ Unintentional disclosure of disease status could occur if other individuals see a patient’s SMS texts,^49^ medication envelopes (for 99DOTS), digital pillbox,^46^ video observation session or adhesive monitor (for ingestible sensors). This may especially be a problem in settings where there are high levels of shared cellphone use within families. Confidentiality may be breached if unauthorised persons access digital adherence data, which may particularly be a problem in settings where regulations for electronic health data are lacking or not enforced. Understanding how cultural characteristics shape patient tolerance for loss of privacy and confidentiality may enable user-centred design. For example, use of a password allowing SMS texts to be read only by the patient may be an option to protect privacy in some settings.^50^

Studies of SMS texting reminders (conducted in Peru, Argentina, Uganda, Pakistan and China),^51^^–^ 52 VDOT (conducted in the USA, Mexico and Kenya),^53–56^ and a digital pillbox (conducted in China)^57^ have generally shown high acceptability of these technologies by patients in surveys and qualitative interviews. Notably, however, one study from the USA suggested low acceptability (33%) of SMS dose reminders, and SMS response rates in actual implementation are often lower (eg, 60%–80% at best) than indicated in acceptability surveys.^52 58^

### Feasibility

Despite widespread cellular ownership in many low-income and middle-income countries (LMICs), there is still considerable variability in levels of cellphone access and cellular network coverage by country as well as potentially within specific subpopulations within countries. As such, feasibility challenges remain in some settings, including the following for cellphone-based strategies: limited cellphone ownership, use of shared cellphones, low cellphone literacy, poor audio or video quality,^59^ poor cellular network connexions, technical failures preventing receipt of SMS texts, electricity outages and changing phone numbers.^49 60^ Feasibility challenges for digital pillboxes may include battery failure, device malfunction (leading to data losses) and problems related to cellular networks. In general, there have been ongoing improvements in the feasibility of DATs. For example, the battery life of digital pillboxes has improved to as long as 6 months. Some devices temporarily store data in flash memory or use GPRS (General Packet Radio Service) to maintain data in transit until acknowledgement of receipt by the web server, reducing data losses due to power failures or poor cellular connectivity.^61^

Surveys conducted in Argentina and China suggested feasibility of SMS-based strategies due to high cellphone ownership and literacy.^62^ ^52^ However, a recent study from Peru highlights that, although cellphone access may be high in the general population, access may be considerably lower in patients who suffer from TB and in particular for patients with TB with poor treatment outcomes.^63^ Implementation studies from high-income and middle-income countries (USA, Canada, Belarus, Mexico) have suggested that VDOT has high feasibility,^53 64–66^ though some patients had to be shifted back to in-person DOT^53^ and low video or audio quality sometimes made dose observation difficult.^65 67^ Ingestible sensors had high feasibility in studies conducted in the USA and Mexico, with >95% of sensor signals detected after ingestion.^37 38^

In some settings, a more fundamental barrier to implementation of DATs may be health system resource constraints, such as lack of computers in clinics to view dosing histories and shortages of HCPs who could act on this information to address non-adherence in high-risk patients.

### Accuracy of digital observation

**Figure 4:**
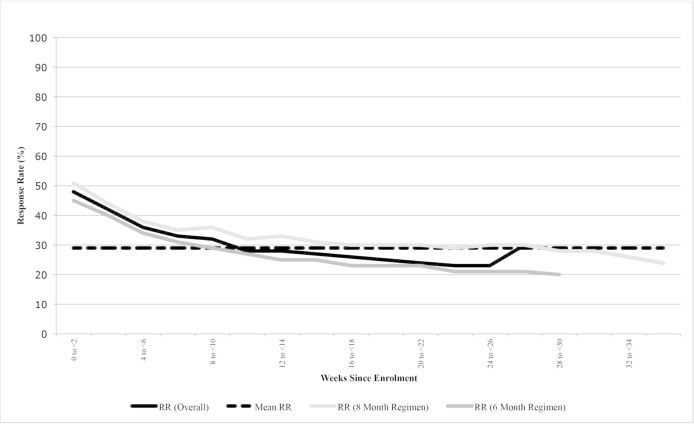
Example of ‘technology fatigue’. Patient response rates to short messaging service (SMS) texts to indicate dose ingestion declined throughout the course of tuberculosis therapy in a study of a two-way SMS intervention in Pakistan, reducing the accuracy of this monitoring approach. Source: Mohammed *et al*.^68^ RR, response rate.

Each DAT has limitations outside of technical failures that may reduce its accuracy for verifying true medication adherence, resulting in over-reporting (ie, false-positive signals in the dosing history) or under-reporting (ie, false-negative signals). Strategies relying on self-reporting via SMS texts or phone calls are at particular risk for under-reporting of adherence if patient engagement wanes due to ‘technology fatigue’, as illustrated in a two-way SMS intervention in Pakistan (figure 4).^68^ Digital pillboxes may under-report adherence if medication blister packs are taken out of the pillbox so that doses can be taken without opening the device or from device non-use due to patient travel or stigma. Ingestible sensors could under-report true adherence if the adhesive monitor used to record signals from the ingestible sensors is removed. Alternatively, over-reporting may occur if patients indicate adherence via SMS texts, phone calls or pillbox openings without taking the doses, though such behaviour often wanes over time.^69^

As such, evaluation of accuracy in research studies is critical to ensure that a DAT provides a reasonable proxy of true adherence. When conducting such research evaluations, careful use of nomenclature is important because it is easy to conflate the quality of a patient’s medication adherence with the quality of her engagement with the DAT, even though these two are not the same. Building on nomenclature recommendations for describing medication adherence,^70^ we suggest parallel nomenclature for describing a patient’s engagement with DATs (table 2).

**Table 2:** Suggested nomenclature for describing medication adherence and engagement with digital adherence technologies

Taxonomy for describing *adherence* to medications (from Vrijens *et al* 70)	Taxonomy for describing *engagement* with digital adherence technologies
Initiation: time point when the first dose of medication is taken	Starting: time point when engagement with technology begins (eg, first SMS text response, phone call, digital pillbox opening etc)
Dosing implementation: correspondence between patient’s actual dosing and the prescribed dosing regimen	Technology participation: correspondence between expected daily engagement with the technology and actual daily engagement
Persistence: length of time between initiation and last dose	Duration of engagement: length of time between starting and stopping of engagement with the technology
Discontinuation: time point when the patient takes her last dose	Stopping: time point when the patient has the last recorded engagement with the technology (eg, last SMS text response, phone call, digital pillbox opening etc)

SMS, short messaging service.

Evaluations of accuracy generally compare DAT-compiled dosing histories to an alternative adherence metric to determine the DAT’s sensitivity, specificity, positive predictive value and negative predictive value for true adherence. These alternative metrics include biological tests of drug ingestion (eg, urine testing for isoniazid content), pill counts, refill data or concurrent monitoring with another DAT, such as use of a ‘silent’ digital pillbox (ie, reminder function disabled) in patients using feature phone-based strategies (online supplementary appendix 1 for details). There is no ‘gold standard’ metric since all comparators, including biological tests, have their own inaccuracies; examples include interindividual and intraindividual variation in drug absorption and metabolism.^71–73^

We recommend using multiple comparators to gain the fullest understanding of a DAT’s accuracy. For example, urine isoniazid testing provides a ‘snapshot’ of dose-taking that can be compared with DAT dosing histories over the prior 24–72 hours, while medication refills provide data on persistence with therapy that can be compared with longer-term dosing histories. Collecting biological test or pill count data during unannounced home visits (ie, without prior notice) may help to minimise the ‘Hawthorne effect’—that is, short-term changes in patient adherence or DAT engagement in anticipation of clinic visits.^74^ In patients concurrently taking medications for other comorbid conditions, such as HIV or diabetes, it is also important to assess the impact of a DAT on medication adherence for all conditions, if possible.

Few studies have rigorously evaluated the accuracy of DATs. One study in China found high correlation between dosing histories from a digital pillbox and urine rifampin test results.^75^ A small pilot study in South Africa found high correlation between dosing histories from digital pillboxes and pill counts conducted for patients taking therapy for MDR TB and HIV.^76^ Studies have suggested that VDOT and ingestible sensors may be more accurate than in-person DOT since they are able to ‘observe’ a greater proportion of doses, especially on weekends and holidays.^38 67^

### Adherence and treatment outcomes

Health outcomes—including medication adherence, treatment success and post-treatment recurrence free-survival—are the most important indicators of DATs’ impact on TB care (table 3, online supplementary appendix 1). When assessing health outcomes, especially in randomised trials or quasi-experimental studies, it is important to identify the appropriate comparator representing the baseline standard of care against which a DAT-based care model will be compared. In many settings, DAT-based models should be compared against DOT, recognising that DOT models may vary from setting to setting, including facility-based DOT, in-person DOT, and community or family DOT (table 1). In other settings, SAT may be the standard of care, though SAT models may also vary based on the frequency of patient–provider contact or medication refills (eg, biweekly, monthly etc). When DAT-based care models are compared against SAT models, we recommend that the SAT model should at minimum include a protocol for patient outreach and engagement if a patient misses a medication refill date since missing a refill may be suggestive of non-adherence. Comparing DAT-based models to SAT models that follow up on missed refills may reveal whether providing HCPs with more granular day-to-day real-time information from DATs (a proposed ‘value-add’ of some of these technologies) actually leads to better outcomes than a more crude but simple approach of following up promptly on patients who miss their medication refill dates.

**Table 3:** Example of information that can be collected to evaluate the impact of medication adherence technologies on treatment outcomes

Outcome	Potential definitions
Medication adherence (ie, dosing implementation and persistence on therapy)	Proportion of all expected doses that were missed during the full treatment course* (continuous outcome)
	Proportion of patient months with >X%† of expected doses missed (continuous outcome)
	Proportion of patients who completed therapy with <X% of expected doses missed over the full treatment course (binary outcome)†
Treatment interruptions	Proportion of patients who completely interrupt tuberculosis (TB) therapy for a short time period (eg, >1 month) or who are formally lost to follow-up (eg, >2 months) (binary outcome)
Treatment success	Proportion of patients who achieved cure or treatment completion (binary outcome)
	Proportion of patients who achieved cure or treatment completion without extension of treatment duration due to non-adherence (binary outcome)
	Mean or median number of medication refills per patient as a proxy of months of treatment completed (continuous outcome)
Post-treatment tuberculosis recurrence-free survival	Proportion of patients who complete TB therapy and achieve 1-year recurrence-free survival (binary outcome)‡§

*An ‘ideal’ length of therapy could be used for assessing the number of expected and missed doses—for example, 182 expected treatment days for patients on daily therapy for drug-susceptible TB (see online supplementary appendix 1).

†Threshold of the percentage of expected doses missed can vary depending on baseline rates of adherence (eg, greater than or less than 10%, 20%, etc).

‡That is, patients who achieve treatment completion and do not experience post-treatment TB recurrence or death.

§Follow-up times can vary, though we recommend a minimum of 6 months of post-treatment follow-up.

While a variety health outcomes may be assessed, improvements in surrogate endpoints that DATs may affect directly (eg, medication adherence) may not always translate into impacts on longer-term outcomes (eg, treatment success or recurrence-free survival) because improvements in longer-term outcomes often require addressing multiple aspects of quality of care, such as ensuring early diagnosis, drug-susceptibility testing, appropriate medication dosing and so on.^77^

As such, we recommend that researchers initially focus on assessing whether DATs improve TB medication adherence. In settings where DATs transform care considerably (eg, shifting away from facility-based DOT), it is also important to routinely monitor treatment success rates to ensure that these outcomes remain comparable, at minimum. When evaluating treatment outcomes, it is important that the entire ‘package of care’ involving the DAT be well defined—including approaches for triaging patients based on dosing histories and the interventions that will be delivered to non-adherent patients.

The study design used to evaluate health outcomes depends on the study goal and resources available. Cohort studies can evaluate whether health outcomes are achieving minimal standards recommended by TB programmes; however, this design is not optimal for understanding whether DATs themselves have contributed to improvements or deterioration in outcomes compared with prior standards of care. Historical programmatic data may be vulnerable to inaccuracy and provide a poor baseline for understanding the relative benefits of DATs.

Quasi-experimental designs that evaluate outcomes prior to and after rollout of a DAT-based intervention may provide more helpful evidence regarding changes in outcomes; however, these findings may be confounded by other concurrent changes to TB care. Trials using randomisation of patients or larger units of care (eg, health facilities) to DAT-based interventions provide the most rigorous evidence of impact on health outcomes.

As highlighted in a recent systematic review,^24^ evidence regarding the impact of DATs on TB outcomes remains limited. Randomised trials of SMS strategies have not shown improvements in adherence or treatment success,^39 52 68 78^ with the exception of a Kenyan study that included prompt engagement by HCPs for patients who did not report pill-taking via SMS.^79^ One South African study of digital pillboxes found improved TB cure rates compared with historical controls, though cure rates in the controls were poor compared with international standards.^80^ A more rigorous cluster-randomised trial in China found that patients in study arms monitored with a digital pillbox had reduction in medication non-adherence (ie, patient-months with >20% of doses missed) compared with the standard of care.^39^ Cohort studies of patients monitored by VDOT in the USA and Australia have shown comparable outcomes to routine in-person DOT.^59 67^

### Costs and cost-effectiveness

In settings with high baseline rates of treatment success, the primary benefit of DATs may be in reduction of costs and patient and HCP burdens associated with existing DOT or SAT models.^9 26 28^ The ‘costs’ of implementation should be defined broadly from a societal perspective to include material components, communication costs, new personnel, changes in time use by existing HCPs, and changes in costs and other burdens borne by patients (table 4). Micro-costing techniques (eg, time and motion studies) may allow estimation of costs resulting from changes in time and work use by HCPs.^81^

**Table 4:** Examples of cost data that should be collected for an evaluation of digital adherence technology-based tuberculosis care delivery

Material costs	Communication costs	Personnel costs	Patient-related costs
Devices if provided to the patient (digital pillboxes, feature phones or smartphones) Platforms for visualising dosing histories by healthcare providers (computers, smartphones) Data servers Medication envelopes (for 99DOTS) Ingestible sensors	SMS text costs Direct phone calls to patients (including call centres for some strategies) Video communication/internet costs	Costs of new personnel for managing information technology or other tasks such as packing medications blister packs in envelopes (for 99DOTS) Cost of new counsellors or other providers in some settings to facilitate more intensive management of barriers to adherence (eg, treatment literacy, depression counselling, treatment of alcohol use disorder) in differentiated care models Changes in resource or time use by existing healthcare personnel due to decreased time spent in direct observation, reduced travel costs with elimination of home DOT, or increased time spent on troubleshooting DATs or managing data entry	Potentially reduced costs of travel and decreased time spent on visiting healthcare facilities for direct observation (under facility-based DOT) Potential time saved by not having wait for a healthcare provider to visit (under home DOT)

DAT, digital adherence technology; DOT, directly observed therapy; SMS, short messaging service.

Estimating an incremental cost-effectiveness ratio requires data on the difference in costs between the existing care model and a DAT-based model, as well as the difference in health outcomes between the two models, ideally represented as disability-adjusted life years. As such, costing studies should collect data on the costs of existing care models (eg, DOT or SAT), and concurrent studies estimating health outcomes for the different models would be required for a formal cost-effectiveness analysis. Modelling may be required to estimate the cost-effectiveness of DATs for TB programmes a national level.^21 82^ Cost-effectiveness of different DATs may vary between high-income countries (where personnel costs are high) and LMICs (where personnel costs are relatively low).

A US study of VDOT factoring in HCP and patient time and travel costs estimated an average cost savings of US$2248 per patient per TB treatment course as compared with home-based DOT^83^; however, an Australian study found that VDOT would be more cost-effective than in-person DOT only with scale-up or decreased technology costs.^59^ A South African study of digital pillboxes estimated a return on investment of 23% over 5 years, largely from cost savings due to improved treatment outcomes; however, this study assumed very poor treatment success rates seen with historical controls.^80^

### Triage strategies and provision of differentiated care

Most DATs aim to identify non-adherent patients who may require additional support to improve adherence or prevent loss to follow-up. Poorly designed interventions for addressing non-adherence may therefore attenuate the beneficial impacts of DATs. HCPs might find themselves in a ‘data glut’, without the guidance or capacity to act productively on the rich real-time data compiled by DATs. A critical question hovering over DAT-based strategies is: how can the data-rich dosing histories compiled by DATs be best leveraged to provide effective individualised (or ‘differentiated’) care that will improve adherence?

Addressing this question will require researchers and HCPs to think beyond the technologies to understand the medical, psychosocial, cultural, structural and health system barriers that contribute to non-adherence.^7^ Such research could inform the development (by iterative testing) of intervention packages targeted towards patients with different levels of risk for non-adherence. Examples of adherence intervention packages that leverage DAT dosing histories exist for other diseases, such as HIV.^21^ These packages of care could screen for and address common causes of non-adherence in each setting, which may include poor treatment literacy,^84^ TB medication toxicities,^5^ depression,^5 85 86^ substance use disorders,^4^ financial burdens^7^ or difficulties travelling to health facilities.^7^ By triaging patients, DATs may allow HCPs to focus more time and attention on a smaller group of high-risk patients, and ancillary personnel, such as dedicated counsellors or psychologists, could potentially help address their more intensive needs. To date, research evaluating triage and differentiated care strategies has been limited.

## Conclusion

DATs have the potential to transform TB care delivery by facilitating more patient-centric strategies for monitoring adherence, while providing HCPs with real-time data that can enable patient triage. The current DAT landscape includes a diverse array of technologies that are in development, undergoing pilot testing or being rolled out at scale as part of clinical care. These DATs employ different devices, reminder functions and approaches for compiling dosing histories, which may contribute to differences in their acceptability, implementation costs and the financial resources required for rollout in different settings.

As such, there is no ‘perfect’ DAT that will work optimally in every setting (especially high-income countries as compared with LMICs) or even for every patient in a single setting. Development of software platforms that can compile dosing histories from multiple DATs may allow HCPs to monitor TB patients who have varied needs using different technologies in the same setting. For example, in India, a single platform has been developed that compiles dosing histories from multiple DATs, which allows patients with cellphones to be monitored using 99DOTS while those without cellphones can be monitored using digital pillboxes. In addition, combining information from DAT-based strategies with data from non-DAT monitoring approaches in clinical practice—such as urine isoniazid testing or medication refill data—may be helpful in cases where patient engagement with the technology is suboptimal, resulting in inaccurate dosing histories.

Research is needed to understand the impact of these technologies on patients and health systems and to inform approaches for provision of differentiated care. Outside of pilot data on VDOT^67^ and ingestible sensors^37 38^ in high-income settings and on digital pillboxes in China and South Africa,^75 76^ little is known about the accuracy of DATs for measuring adherence in patients with TB, especially with larger-scale implementation in LMICs and for patients concurrently taking medications for comorbid conditions such as HIV or diabetes. Most importantly, more robust data are required on DATs apart from two-way SMS to determine whether they have positive impacts on health outcomes, especially in high-TB-burden LMICs.

Finally, while studies in TB have heavily focused on the DATs themselves, less emphasis has been placed on understanding how DATs can be leveraged to provide differentiated care to patients who require more intensive support to achieve optimal treatment outcomes. Little work has been conducted to understand the causes of medication non-adherence in different populations of patients with TB, so that clinical protocols can be designed to help HCPs screen for and address these causes. If the rollout of DATs also stimulates rethinking of the HCP–patient interaction, then DATs have the potential to move ‘care’ into non-traditional spaces (such as the home or the workplace) and to serve as an extension of the health system.^32^ Otherwise, DAT-based monitoring strategies run the risk of overly focusing on ‘observation’ and replicating some of the paternalistic aspects of existing DOT approaches. In our opinion, if DATs are viewed as tools for enhancing (rather than limiting) face-to-face human interactions, then they will have stronger potential for transforming TB care delivery by creating truly patient-centric models of care.
