# Supplementary material for: Digital adherence technologies for the management of tuberculosis therapy: mapping the landscape and research priorities
Source: BMJ Glob Health. 2018 Oct 11;3(5):e001018. doi: 10.1136/bmjgh-2018-001018 (PMC6195152; doi:10.1136/bmjgh-2018-001018)
Supplement: Supplementary data [file bmjgh-2018-001018supp001.pdf]

SUPPLEMENTARY FILE

Supplement to:

Digital adherence technologies for the management of tuberculosis therapy: mapping the landscape and research priorities

Ramnath Subbaraman, MD; Laura de Mondesert, MPH; Angella Musiimenta, PhD; Madhukar Pai, PhD; Kenneth H. Mayer, MD; Beena E. Thomas, PhD; Jessica Haberer, MD

Corresponding Author:

Ramnath Subbaraman, MD, MSc, FACP
Department of Public Health and Community Medicine, Tufts University School of Medicine, and Division of Geographic Medicine and Infectious Diseases, Tufts Medical Center, Boston, USA
136 Harrison Avenue, MV237
Boston, MA 02111
Tel: +1-617-636-6807
Email: ramnath.subbaraman@tufts.edu

Appendix 1. Description of digital adherence technologies

Short messaging service (SMS)-based strategies

Description

Short messaging service (SMS) texting is a potentially cost-efficient approach to facilitating patient-health system engagement throughout TB therapy, due to the relatively ubiquitous and rapidly increasing access to mobile phones globally, including in low- and middle-income countries. SMS-based interventions have been employed alone[1,2] and in combination with other DATs.[3,4]

Patients are sent a daily motivational text message at a pre-specified time, encouraging them to take their medications. In most approaches, messages are written in a neutral, non-discriminatory voice in the patient's preferred language and make no mention of TB due to the social stigma associated with the disease.[1,5] Some approaches purposefully vary message content and design the messaging strategy using motivational concepts from behavioral economics.[2] Patients report taking the day's dose by responding to the reminder via a free SMS text or by placing a toll-free call.[1,2] If the patient does not respond after the first reminder text (e.g., within 2 hours), additional SMS text reminders may be sent each day to the patient until a response is received.[1,2]

HCPs are notified when the system does not receive a text response from a patient, which may suggest a missed dose. In an approach used in Kenya, HCPs intervened the same day that a dose had potentially been missed through individualized SMS texts to the patient or through a phone call.[2] In an approach used in Pakistan, HCPs were encouraged to contact patients who have not engaged with the system after seven days with a phone call.[1]

Approach to digital observation

A SMS text or phone call from the patient is used as a proxy for a dose taken. However, this approach to digital observation does not confirm that a dose was actually ingested. In addition, the patient effort required to report with this model has generally resulted in under-reporting of pill-taking, and this under-reporting may get worse throughout the course of therapy due to "technology fatigue."[1,2]

Potential benefits and limitations of the technology

SMS texts provide a relatively low-cost approach for providing regular dose reminders to patients, and these reminders can be implemented in conjunction with other DATs.[3,6] Two-way SMS also allows monitoring of medication adherence based on responses to SMS reminders, and automated triage systems can be designed around these adherence data. However, due to the effort involved in sending a SMS response, patient motivation to report adherence may decline over time.[1,2] Problems with cellular reception, service or carrier compatibility, and mobile phone functionality may also affect the success of two-way SMS services in some settings. Some rural settings with poor commercial cellular coverage may require general packet radio service (GPRS) networks to transmit cellular signals, and SMS-based strategies may not be feasible in some rural areas without GPRS coverage.[7]

Relevant studies

A study of a mixed population of patients in the USA, including many with latent TB, found that a high proportion (77%) owned cellphones but a considerably lower proportion (33%) felt that SMS text reminders for medication adherence would be acceptable.[8] In contrast, all patients with TB in small focus group discussions in Peru and the vast majority surveyed in studies in Uganda and China (96% and 80% respectively) felt that SMS text reminders to support medication adherence are acceptable or

"helpful".[9-11] Studies in Argentina and Pakistan also found two-way SMS approaches to have reasonable patient acceptability, though SMS response rates in implementation were roughly 60%—80% at best.[12,13]

A quasi-experimental study in Uganda found no improvement in TB treatment completion rates with a one-way SMS medication reminder intervention compared to historical controls, though the study was underpowered.[10] In contrast, a randomized trial of a one-way SMS intervention in China found significant improvements in treatment completion rates and reductions in treatment interruptions and missed medication doses.[14] In a randomized controlled trial conducted in Pakistan, the use of two-way SMS texts did not improve the rate of TB treatment completion or self-reported adherence, when compared to patients who did not receive two-way SMS texts.[1] The study did not discuss how cellular reception issues may have affected the accuracy of patient reporting.[15] A study in China similarly found that SMS text message reminders did not improve TB medication adherence unless used in combination with adherence monitoring using an digital pillbox.[3] A study in Kenya found that a two-way SMS intervention significantly improved TB treatment completion rates.[2] In contrast to the studies in Pakistan and China, the Kenyan study incorporated routine variations in SMS text content, and non-response to multiple reminder SMS texts prompted outreach the same day by a HCP. A blinded randomized controlled trial of SMS reminders is currently underway among TB patients in Cameroon.[5] A randomized trial currently underway in Lesotho among TB patients co-infected with HIV is implementing a one-way SMS strategy as part of a combination intervention package that includes in-person interventions by nurses and community health workers using a standardized clinical algorithm;[16] the findings of this trial may provide insights into the optimization of outreach by HCPs as part of differentiated care.

99DOTS

Description

99DOTS was developed by researchers at Everwell Health Solutions as a low cost approach for tracking TB medication adherence using a combination of custom medication packaging and cellphones (Figure 1).[4,6] To date, 99DOTS has been rolled out to nearly 45,000 patients in India's public sector TB program. During registration for TB treatment, HCPs counsel a patient about using 99DOTS and collect up to three cellphone numbers from a patient for registration in the 99DOTS system. Upon registration, a patient chooses a time of day when she would like to receive a free SMS text reminding her to take her dose.

Patients are issued TB medications in blister packs wrapped in custom envelopes. Dispensing a pill breaks a perforated flap on the envelope, revealing a toll-free phone number (unpredictable to the patient) that the patient dials, leaving a "missed" (unanswered) call that gets registered by a computer server as a dose that was dispensed (a proxy for medication ingestion). In addition to a routine SMS reminder to take the day's dose, failure to call on a given day prompts the system to send a repeat SMS reminder to the patient. HCPs receive SMS notifications regarding non-adherent patients, and the 99DOTS server automatically triages patients into different risk categories depending on the number of doses missed. HCPs can also monitor a patient's dosing history in real time, through a web portal accessible via computer or smartphone. HCPs are supposed to contact non-adherent patients via phone or home visits to provide enhanced counseling and address underlying causes of medication non-adherence.

The costs of implementing 99DOTS are generally low in LMIC settings (approximately $5 to $6 per treatment course). However, the costs of implementing 99DOTS may vary depending on the scale of deployment, the in-country telecommunications offerings, the number of phone numbers used for

reporting doses, usage of missed calls versus interactive voice response calls for reporting doses, usage of toll-free versus non-toll-free calls, the size of medication blister packs (which affect envelope size and printing costs), the strategy used for wrapping drugs, and the level of technical and non-technical support offered by organizations facilitating roll-out, such as Everwell Health Solutions.

Approach to digital observation

99DOTS probably has greater specificity for confirming doses taken than two-way SMS approaches. When registering a “dose taken,” the 99DOTS computer system is able to recognize both the cellphone from which the patient is calling (since it was registered at the time of treatment initiation) and the dose-associated phone number dialed by the patient, thereby attributing a specific dose taken to a specific patient. In cases where a patient forgets to dial in a dose but a HCP is able to verbally confirm that the dose was taken, HCPs can manually report these doses to the 99DOTS system. The 99DOTS dosing history dashboard differentiates these HCP-verified doses from doses dialed-in directly by patients.

The 99DOTS approach does not confirm that a dose was actually ingested. In theory, a patient could dispense a dose and call the associated phone number without actually swallowing the medications. In addition, the patient effort required to report with this model may lead to under-reporting of pill-taking, especially later in the treatment course due to “technology fatigue.” Finally, while manual reporting of doses by HCPs may document a more complete dosing history, it could theoretically allow for falsification of adherence records by HCPs.

Potential benefits and limitations of the technology

99DOTS is relatively inexpensive, since patients and HCPs use their own feature- or smart-phones to call in doses and access dosing histories, respectively. Most of the cost of this approach is attributable to printing of the custom paper envelopes for the medications. Unlike technologies such as video DOT, 99DOTS allows patients to retain some anonymity when reporting pill-taking. The 99DOTS HCP interface allows providers to evaluate and track both individual patient adherence and the progress of an entire treatment program in real-time. 99DOTS' triage function allows HCPs to prioritize time and resources to patients who may have poor medication adherence, and providers can opt to receive more frequent alerts for high-risk patients they would like to follow more closely.

As with two-way SMS approaches, reporting pill-taking requires some patient effort with 99DOTS, and patient motivation to report adherence may decline over time. 99DOTS may also be difficult to implement in locations with limited mobile phone availability, cellular service or carrier compatibility issues, and problems with cellular reception, especially in rural settings with poor commercial cellular and GPRS coverage.[7]

Relevant studies

An observational study evaluating the acceptability of 99DOTS to patients and providers is currently underway in India. The study will also assess the accuracy of 99DOTS for measuring medication adherence as compared to urine tests for isoniazid content collected from patients during unannounced home visits.

Video Directly Observed Therapy (VDOT)

Description

Video (or virtual) DOT (VDOT) is the DAT that most closely mimics standard DOT while still allowing patients to take their medications at home or another preferred location. HCPs observe patients take their TB medications via videoconferencing over a computer webcam or smartphone, eliminating the need for patients to travel to a clinic or for HCPs to travel to patients' homes (Figure 1).[17]

In practice, VDOT has employed two different strategies: synchronous and asynchronous VDOT. In synchronous VDOT, live-streaming technology connects patients with HCPs in a pre-scheduled videoconferencing call, where HCPs watch patients ingest medications in real time.[18,19] Patients may receive SMS text reminders about upcoming or missed conference calls.[18] When patients miss conference call appointments or if there are challenges with video connectivity, HCPs follow-up patients with phone calls or home visits for especially non-engaged patients.[18]

Asynchronous VDOTs allow patients to record themselves ingesting the medication dose using a webcam or smartphone.[20] Patients submit these recordings to a secure website where HCPs can view the videos at their convenience to observe adherence. Missed VDOT submissions may be followed up by reminder SMS texts, phone calls, or, if unsuccessful, home visits.[20] In one variation of asynchronous VDOT, computer-automated facial recognition, medication identification, and ingestion confirmation may eliminate the need for HCPs to spend time watching these videos to confirm that a dose was taken, which is an approach currently being pilot-tested in the USA.[21]

Approach to digital observation

In most VDOT approaches, patients are instructed to name and show each pill to the camera before swallowing it and opening their mouths to demonstrate the pill was ingested.[18] As such, VDOT provides

a fairly rigorous verification of medication ingestion, though concealment of medications inside the mouth without swallowing it is theoretically possible. As with two-way SMS and 99DOTS, reporting pill-taking requires patient effort, arguably more so than with the other two strategies. Patient motivation to show up to pre-scheduled conference calls (with synchronous VDOT) or to submit videos (with asynchronous VDOT) could wane over time due to "technology fatigue," which could result in under-reporting of true adherence. In addition, falsification of patient adherence records by HCPs is theoretically possible with this approach, which could result in over-reporting of adherence, though computer-automated approaches could limit this theoretical risk.

Potential benefits and limitation of the technology

In contrast to 99DOTS and two-way SMS, VDOT may facilitate monitoring of patients with complex TB medication regimens involving multiple tablets (e.g., MDR patients) or patients who are also taking medications for other comorbidities (e.g., HIV or diabetes). In contrast to most other DATs, synchronous VDOT allows for daily patient-provider interactions in real time, which allows patients to discuss other aspects of their care, such as medication adverse effects.[18] Anecdotal evidence suggests that VDOT may also help providers identify if patients are taking incorrect medication regimens, as HCPs are directly visualizing the medications being ingested. The pre-scheduled nature of synchronous VDOT conference calls may be limiting for some patients, however. While asynchronous VDOT does not allow for discussion of patient concerns in real time, it does allow patients to take their medications at a time and location most convenient to them.[20] They can still report problems in their care, such as medication adverse effects, via the recorded video, though HCPs cannot address these concerns in real time. Computer-automated "observation" may further reduce potential for patient-provider interactions.[21,22]

In addition to potential for "technology fatigue," some patients may perceive VDOT as being invasive or of violating confidentiality. VDOT also generally requires that patients have smartphone or computer webcam access, higher-quality cellular service than is required for SMS or 99DOTS, or high-speed Internet connections that allow for video-conferencing. Patients also need to have smartphone or computer literacy.[18] These requirements may limit the rate of scale-up in LMICs or even in some high-income country settings.[19]

Relevant studies

Qualitative and patient survey-based studies of VDOT conducted in Illinois (USA),[20,23] the US-Mexico border,[24] and Kenya[25] all showed high (though not universal) acceptability among patients and all studies showed near-universal patient preference for VDOT over traditional DOT models. Studies from high- and middle-income countries—including the USA, Canada, Belarus, and Mexico—generally show that VDOT has high feasibility,[17,19,20,26] though some patients had to be shifted back to standard in-person DOT[20] and technical problems sometimes resulted in low picture quality or poor audio connectivity, making dose observation difficult.[18,19]

A study conducted in Washington state (USA) found that VDOT may yield an average cost-savings of US $2,248 per patient over the entire TB treatment course as compared to home-based DOT, [27] and an Australian study found that VDOT could be more cost-effective than traditional forms of in-person DOT only if expanded on a larger scale or with decreased technology costs.[28] A Canadian study found that VDOT visits were 26 minutes shorter on average than home-based DOT visits.[19] A cohort study conducted in New York (USA) found that VDOT resulted in similar treatment completion rates, a greater proportion of "observed" medication doses, and increased HCP efficiency when compared to home-

based DOT.[18] Randomized trials of VDOT versus standard DOT approaches are currently underway in the USA,[21] Moldova[29] and London.[30]

Digital pillboxes

Description

Digital pillboxes were designed to monitor adherence to TB treatment by recording pillbox openings and closings as proxy indicators of doses taken, which allows patient adherence to be monitored without in-person or video observation by providers.[31] Digital pillboxes may also include a daily reminder function that can be programmed for different times of the day, based on patient preference. Depending on the type of pillbox, these reminders present as some combination of visual displays (e.g., a glowing light), alarms, and automated voice alerts integrated into the device (Figure 1).[3,31,32] With most digital pillboxes, patients start receiving reminders if the device is not opened within a predefined time window, and they stop receiving reminders for the day after the box has been opened that day.[3] In some digital pillbox-based strategies, SMS text reminders may also be sent to the patient's cellphone.[3]

Using a sensor, the digital pillbox records when a patient opens and closes the box, presumably to take a medication dose. In remote real-time monitoring approaches, the pillbox's SIM card transmits this information to a server on regular (daily or semi-weekly) basis, which allows compilation of a medication dosing history in real time. HCPs access this information on a web-based interface on a computer or smartphone and can intervene upon medication non-adherence before the patient's next clinic visit. Some digital pillbox-based strategies can also be designed to alert providers in real time via SMS when patients have missed prescribed doses. In clinic visit-based monitoring approaches, HCPs transfer data

on openings and closings of the digital pillbox directly from the device during clinic visits, by uploading the dosing history from the pillbox to a computer or smartphone using a USB port.[32] While this approach does not allow for real-time intervention, HCPs can use the dosing history compiled since the patient's last visit to provide enhanced adherence counseling, as was done during a recent study in China.[3] To help differentiate between a patient not opening the pillbox on a given day and pillbox malfunction, some digital pillboxes record or transmit a daily "heartbeat" signal, which indicates that the device is functioning and has adequate battery life remaining.[32]

Approach to digital observation

As described above, opening and closing an digital pillbox serves as a proxy indicator of adherence. In theory, the act of digital observation of a dose does not involve any extra effort outside of what is normally required by a patient to take her medications, which is in contrast to other DATs, especially feature- or smart-phone-based strategies.

There are potential limitations to this approach to digital observation, however. As with two-way SMS and 99DOTS, digital pillboxes cannot verify that a patient actually ingested the medication, which may be especially problematic with digital pillboxes because some patients may quickly open and close the pillbox just to shut off repeated reminders. In addition, especially with larger digital pillboxes, patients may take medication blister packs out of the device, for example when traveling or going to work. This could result in under-reporting of medication adherence.

Potential benefits and limitation of the technology

In addition to the relative ease of digital observation with digital pillboxes, these devices have a few other potential benefits. Digital pillboxes may have room on the device for placement of medication

instructions that may help to educate patients about correct pill taking. Unlike other DATs, for which reminders are generally sent via SMS text to a patient's cellphone, the reminder function with digital pillboxes is integrated with the site where the medications are actually stored. Medications for multiple diseases (e.g., TB and HIV) can be stored and monitored using a single pillbox. Given the complexity of MDR TB therapy, digital pillboxes have strong potential to help patients with the organization, storage, and monitoring of therapy for this form of TB.

Digital pillboxes also have some potential limitations. Digital pillboxes require that patients be provided with an additional device outside of their own feature- or smart-phone. As a result, digital pillboxes may be relatively more expensive than other strategies for monitoring TB medication adherence, especially feature phone-based strategies, which may limit their use by some LMIC TB programs. Digital pillboxes may be more visible objects for medication storage (as compared to blister packs, for example), which could be a concern for patients worried about confidentiality and TB-related stigma. Some digital pillboxes may be bulky and difficult to travel with, as noted above. Finally, as with other DATs, real time digital pillbox-based strategies may be difficult to implement in locations with limited mobile phone availability, cellular service or carrier compatibility issues, and problems with cellular reception, especially in rural settings with poor commercial cellular and GPRS coverage.[7] Non-real time clinic visit-based monitoring approaches may still be feasible in these settings.[3]

Relevant studies

In rural China, a study evaluating the feasibility and acceptability of an digital pillbox-based monitoring strategy found relatively high performance, acceptability, and satisfaction with the technology among TB patients and HCPs; however, HCPs had challenges in operating the software platform for visualizing adherence data and patients had difficulties understanding the medication labeling information inside

the pillbox.[32] Evaluations of the feasibility and acceptability of digital pillbox-based monitoring strategies are also currently underway in Uganda (focused on patients with drug-susceptible TB)[33] and India (focused on patients with MDR TB).

A randomized trial in China compared a two-way SMS-based strategy, a digital pillbox-based strategy, and a combined strategy that included both technologies. The study found no improvement in poor medication adherence with two-way SMS, a 42% reduction in poor adherence with the digital pillbox alone, and a 51% reduction in poor adherence with the combined strategy.[3] A randomized trial evaluating an digital pillbox-based monitoring strategy's impact on TB recurrence-free survival is currently underway in China.[34]

Ingestible sensors

Description

Ingestible sensors have the potential to record actual medication ingestion, as opposed to the proxy indicators of ingestion measured by other DATs—such as pillbox opening and closing in the case of digital pillboxes or dispensing of a specific dose from a blister pack in the case of 99DOTS. Ingestible sensors are tiny microchips imprinted with unique barcodes that can be embedded in medications. Upon pill ingestion, the pill's capsule dissolves and the embedded sensor separates from the capsule, allowing gastric fluids to react with the sensor. This reaction forms an electric field that transmits the barcode to an adhesive monitor (a patch) worn on the patient's skin.

The adhesive monitor records information from the barcode regarding the time and date of pill ingestion.[35] The adhesive monitor wirelessly transmits pill-taking information to the patient's smartphone, which can then transmit adherence information to a server via cellular networks to allow HCPs to access dosing histories on a computer dashboard.[35,36] This dashboard allows providers to follow a patient's medication adherence in real-time. Since current ingestible sensor-based monitoring approaches require that the patient have a smartphone, daily medication reminders can be sent via SMS texts to patients.[36] Both the ingestible sensor and the adhesive monitor are considered to be safe for patient use.[35]

Approach to digital observation

As described above, the transmitted signal from the ingestible sensor after its interaction with gastric juices serves as the indicator of medication adherence. Potential advantages of this approach may include improved precision in confirming whether and when a pill was actually taken. This precision is in contrast to SMS-bases strategies, 99DOTS, and digital pillboxes, in which proxy indicators of medication ingestion with each technology cannot actually confirm whether a dose was swallowed. In addition, the act of digital observation of a dose does not entail extra effort outside of what is normally required by a patient to take her medications.

However, there are potential limitations to the accuracy of ingestible sensors. The bioelectric signal transmitted by an ingestible sensor upon contact with gastric juices is only active for about seven minutes. If the patient's monitor is poorly adhesive to the patient's skin or if the patient removes the monitor, the signals transmitted by ingestible sensors cannot be recorded, which may result in under-reporting of medication adherence.[37]

Potential benefits and limitations of the technology

Ingestible sensors enable HCPs to monitor actual medication ingestion with relative precision. For patients, the effort involved in “reporting” medication adherence is relatively limited, assuming that they are willing to wear the adhesive monitor and carry a functional smartphone. Ingestible sensors also eliminate the need for patients to learn how to use a new piece of technology.

Some patients may have concerns about swallowing ingestible sensors, even though the sensors have been shown to be associated with minimal adverse effects.[35,36] Patients may feel that this form of monitoring is overly invasive. Patient acceptability could therefore serve as a barrier to use in some settings and is an important priority for research. As with other DATs, ingestible sensor-based strategies may be difficult to implement in locations with limited smartphone availability, cellular service or carrier compatibility issues, and problems with cellular reception, especially in rural settings with poor commercial cellular and GPRS coverage.[7] Finally, feasibility, accuracy, and cost data are required in LMIC contexts to determine whether ingestible sensors have potential applicability in these settings.

Relevant data

Ingestible sensors have raised concerns about the ethics and acceptability of this level of surveillance in the academic literature and the popular press.[38,39] A small feasibility study involving 30 TB patients conducted at two sites in the USA found that 95% of sensors ingested by patients were successfully detected and three false signals (i.e., doses recorded even though sensors were not ingested) were registered by the adhesive monitors. Four adverse effects—mild skin rashes at the site of the adhesive monitor and nausea—were deemed to be possibly related to the monitoring approach.[35] Another feasibility study involving 75 TB patients conducted in Mexico found that 99% of doses with ingestible sensors that were directly observed by a HCP were also correctly recorded by the adhesive monitor and

transmitted to the monitoring dashboard. Ingestible sensors were able to confirm more doses than in-person DOT. Three percent of patients developed a local rash at the site of the adhesive monitor.[36] In a modeling exercise, an ingestible sensor-based monitoring strategy was estimated to have lower costs for public health clinics and patients as compared to different in-person DOT strategies in a US clinical setting.[40]

Appendix 2. Details of methodological approaches for evaluating the accuracy and health outcomes associated with use of digital adherence technologies (DATs)

Accuracy of digital observation

There are multiple potential comparator tests that can be used to evaluate the accuracy of DATs. These include the following:

Biological tests of medication ingestion: Testing a patient's urine for isoniazid content is the most common biological test of TB medication ingestion used in most studies.[41-44] Two validated point-of-care urine tests for isoniazid are commercially available (IsoScreen® and Taxo INH®).[44-46] Test characteristics are not significantly affected by whether a patient is a slow or fast acetylator of isoniazid.[46] Nearly all patients who take isoniazid have a positive urine test at 6 to 24 hours after medication ingestion.[46,47]

However, urine isoniazid testing is not without limitations. Isoniazid has a prolonged clearance time, with 100%, 83%, 28%, and 11% of patients having positive urine tests at 36, 48, 60, and 72 hours after medication ingestion based on pooled results from two studies.[46,47] Therefore, urine isoniazid is most sensitive for identifying non-adherence for ≥72 hours for patients on daily therapy, and it is most likely to test positive for patients who took a medication dose within 6 to 48 hours of testing.

Even though urine rifampin testing was used as the comparator metric in one study of a DAT,[48] there are substantial limitations to this test for a few reasons. First, pharmacokinetic studies suggest that rifampin is

cleared from plasma (and therefore not present in urine) in <12 hours in many patients.[49] Second, three prior studies utilizing urine rifampin to evaluate TB medication adherence found this test to be inaccurate, due to its rapid urinary clearance and the poor reliability of urine color change for test interpretation.[44,45,50] Third, per our knowledge, commercial tests for urine rifampin are not widely available.

Pill counts: Pill counts provide information on a patient's medication adherence at least since the last refill date. Evaluating medication adherence during pill counts during clinic visits has the potential to overestimate adherence due to the risk of "pill dumping" by patients immediately before the visit due to the social desirability of appearing adherent. As such, pill counts conducted during unannounced home visits may have greater accuracy than clinic-based pill counts. In some cases, unannounced pill counts have been found to yield comparable adherence information to real-time monitoring using digital pillboxes in patients with HIV,[51] though clinic-based pill counts were found to have relatively poor sensitivity for detecting non-adherence in TB patients in Tanzania.[52] In addition, data from some HIV trials suggest pill count data may have no better accuracy than self-reported adherence.[53] In some contexts, pill counts could be considered to be invasive by patients. While unannounced pill counts conducted via phone call have been found to have similar reliability to unannounced pill counts conducted via home visits in some high-income country contexts,[54] a study conducted in Lesotho and Ethiopia found this approach to have low feasibility, because patients often had their phones turned off or were away from their pills when called.[55]

Medication refill visits: Delays in medication refill visits may be suggestive of medication of non-adherence, because patient would likely have run out of pills if he or she obtains a late refill. This information provides relatively crude information regarding true adherence and is therefore not an ideal comparator for evaluating the accuracy of DATs. Medication refill visit information is more helpful for

measuring the longer-term accuracy of DATs. For example, patients enrolled in feature phone-based adherence monitoring strategies may stop sending SMS texts or phone calls to report medication adherence at some point during the treatment course. If these same patients continue to pick up their medication refills on time, this provides valuable information suggesting that patients may be experiencing "technology fatigue."

Concurrent monitoring with another DAT: Concurrent monitoring with another DAT can sometimes help to assess the accuracy a DAT of interest. For example, the accuracy of 99DOTS could be evaluated by placing the medication blister packs into a "silent" digital pillbox (i.e., with a disabled audiovisual reminder) that records when the blister packs are accessed based on openings and closings of the digital pillbox, providing an alternative adherence record that can be compared to the 99DOTS dosing history compiled through patient phone calls. "Silent" digital pillboxes were used in this manner to evaluate whether SMS text messaging reminders improved adherence in China[3] and to assess the accuracy of a variety of alternative adherence metrics in Tanzania.[52]

Adherence and treatment outcomes

We discuss in detail potential treatment outcome measures that can be used for assessing medication adherence (i.e., the quality of dosing implementation), persistence (i.e., length of time) on therapy, treatment success, and post-treatment TB recurrence-free survival.

Medication adherence and persistence on therapy: Medication adherence, or the quality of dosing implementation, can be thought of as the proportion of expected doses actually taken by a patient in a given time period. Persistence on therapy can be thought of as the total length of time that a patient is

on therapy, regardless of the quality of dosing implementation during that time period. Both of these measures are probably related to the risk of disease relapse in TB.[56-58]

Using either of these indicators alone as a study outcome may provide an incomplete picture of the impact of DATs on patient behavior and potential longer-term TB outcomes. For example, a TB patient could in theory have perfect dosing implementation for the first few weeks of therapy but subsequently be lost to follow-up and therefore have poor persistence on therapy. A TB patient could alternatively miss numerous doses of TB medications but persist in completing a full length of therapy or even extended course of therapy.

As such, we recommend that metrics that integrate these two indicators should ideally be used as outcome measures in studies of DATs. Table 2 in the main manuscript provides examples of metrics that integrate persistence and adherence. For example, researchers could measure the total number of doses taken during the first 182 days of therapy for patients taking daily therapy for drug-susceptible TB, since 182 total doses of medication are often recommended to complete a full course of treatment, though recommended durations may vary in different countries.[59] A TB patient who takes her medications perfectly would finish all of these doses within the first 182 days. Therefore, taking fewer than 182 doses in the first 182 days would highlight patients who had suboptimal adherence (i.e., some missed doses during this time period) and patients who had suboptimal persistence (i.e., discontinuation of therapy during this time period). The other metrics proposed in Table 2—such as the number of patient-months in which a TB patient met a predefined adherence threshold—also integrate information on both adherence and persistence.

Treatment success: Standard case definitions for TB treatment outcomes—i.e., "cure," "treatment completion," "treatment failure," "loss to follow-up," etc.—have been outlined by the WHO.[60] Treatment success, which represents the sum of patients who achieve cured or complete therapy, is commonly used as a study outcome in TB research. However, for studies of DATs and medication adherence more generally, treatment success alone as a study outcome could be misleading, because treatment for TB patients is often extended until a patient takes a minimum number of medication doses (e.g., 182 doses for drug-susceptible TB patients).[59] Patients in some settings may have substantial extensions of TB therapy for missed medication doses, sometimes by as much as three or four months.[56] One study in India found a strong linear association between the length of TB treatment extension and post-treatment disease relapse rates.[56] Since extended treatment courses in many TB programs reflect missed medication doses and suboptimal adherence, evaluating the "proportion of patients who achieved cure or treatment completion without extension of treatment duration" may serve as a modified metric for treatment success that also reflects the quality of patient's medication adherence (Table 2).

Post-treatment TB recurrence-free survival: Post-treatment TB recurrence-free survival as assessed 12 months after completing TB therapy is a recommended clinical efficacy endpoint for drug trials by regulatory bodies such as the U.S. Food and Drug Administration (FDA).[61] Post-treatment TB recurrence may be a particularly insightful outcome for trials of DATs, because the quality of a patient's medication adherence is strongly associated with the risk of disease relapse.[56] This may however, be a less optimal outcome to use in settings with a high prevalence of HIV co-infection, because a larger proportion of TB recurrences may be due to reinfection with a new TB strain rather than relapse of the strain that previously caused active TB disease.[62,63] We recommend following patients for at least 12 months after treatment completion to determine disease recurrence rates, based on the findings of meta-analysis

that found that most TB recurrences (91%) occur within one year of treatment completion.[64] We also suggest that evaluations of post-treatment disease recurrence include post-treatment deaths as an adverse outcome (in addition to disease recurrence) and that patients should be closely followed up with phone calls or home visits every few months to prevent loss to follow-up, so that all outcomes can be clearly captured, especially since routine clinical records may be incomplete. In evaluating post-treatment recurrence rates, any symptoms that may be suggestive of TB recurrence should be carefully evaluated, ideally with mycobacterial culture for patients who previously had pulmonary TB,[56] given the lack of specificity of sputum microscopy or Xpert MTB/Rif as tests for detecting disease relapse.

References

1. Mohammed S, Glennerster R, Khan AJ. Impact of a Daily SMS Medication Reminder System on Tuberculosis Treatment Outcomes: A Randomized Controlled Trial. *PLoS One* 2016; **11**(11): e0162944.

2. Yoeli E, Rathauser J, Rand D. Self-verification and behavioral interventions via mobile phones drastically improves tuberculosis treatment success in a randomized control trial (abstract OA-136-12). In: Proceedings of the 48th Union World Conference on Lung Health [Internet]; 2017 Oct 11-14; Guadalajara, Mexico. Guadalajara: The Union; 2017 [cited 2018 Mar 15]; p. S65. Available from: http://www.abstractserver.com/TheUnion2017/TheUnion2017_Abstracts_Web.pdf.

3. Liu X, Lewis JJ, Zhang H, et al. Effectiveness of Electronic Reminders to Improve Medication Adherence in Tuberculosis Patients: A Cluster-Randomised Trial. *PLoS Med* 2015; **12**(9): e1001876.

4. Oberoi S, Kumar Gupta V, Neha C, Singh A. 99 DOTS Mini-Review. *International Journal of Contemporary Medical Research* 2016; **3**(9): 2760-62.

5. Bediang G, Stoll B, Elia N, et al. SMS reminders to improve the tuberculosis cure rate in developing countries (TB-SMS Cameroon): a protocol of a randomised control study. *Trials* 2014; **15**: 35.

6. Cross A, Kumar M, Soren P, et al. 99DOTS: monitoring and improving TB medication adherence using mobile phones and augmented packaging (abstract OA-414-05). In: Proceedings of the 46th Union World Conference on Lung Health [Internet]; 2015 Dec 2-6; Cape

Town, South Africa; Cape Town: The Union; 2015 [cited 2018 Mar 15]; p. S288. Available from: http://capetown.worldlunghealth.org/Abstract_Book_2015-Web.pdf.

7. Siedner MJ, Haberer JE, Bwana MB, Ware NC, Bangsberg DR. High acceptability for cell phone text messages to improve communication of laboratory results with HIV-infected patients in rural Uganda: a cross-sectional survey study. *BMC Med Inform Decis Mak* 2012; **12**: 56.

8. Person AK, Blain ML, Jiang H, Rasmussen PW, Stout JE. Text messaging for enhancement of testing and treatment for tuberculosis, human immunodeficiency virus, and syphilis: a survey of attitudes toward cellular phones and healthcare. *Telemed J E Health* 2011; **17**(3): 189-95.

9. Albino S, Tabb KM, Requena D, et al. Perceptions and acceptability of short message services technology to improve treatment adherence amongst tuberculosis patients in Peru: a Focus Group Study. *PLoS One* 2014; **9**(5): e95770.

10. Hermans SM, Elbireer S, Tibakabikoba H, Hoefman BJ, Manabe YC. Text messaging to decrease tuberculosis treatment attrition in TB-HIV coinfection in Uganda. *Patient Prefer Adherence* 2017; **11**: 1479-87.

11. Lei X, Liu Q, Wang H, Tang X, Li L, Wang Y. Is the short messaging service feasible to improve adherence to tuberculosis care? A cross-sectional study. *Trans R Soc Trop Med Hyg* 2013; **107**(10): 666-8.

12. Mohammed S, Siddiqi O, Ali O, et al. User engagement with and attitudes towards an interactive SMS reminder system for patients with tuberculosis. *J Telemed Telecare* 2012; **18**(7): 404-8.

13. Iribarren S, Beck S, Pearce PF, et al. TextTB: A Mixed Method Pilot Study Evaluating Acceptance, Feasibility, and Exploring Initial Efficacy of a Text Messaging Intervention to Support TB Treatment Adherence. *Tuberc Res Treat* 2013; **2013**: 349394.

14. Xue-Hui Fang, Shi-Yang Guan, Li Tang, et al. Effect of Short Message Service on Management of Pulmonary Tuberculosis Patients in Anhui Province, China: A Prospective, Randomized, Controlled Study. *Med Sci Monit* 2017; **23**: 2465-9.

15. DiStefano MJ, Schmidt H. mHealth for Tuberculosis Treatment Adherence: A Framework to Guide Ethical Planning, Implementation, and Evaluation. *Glob Health Sci Pract* 2016; **4**(2): 211-21.

16. Howard AA, Hirsch-Moverman Y, Frederix K, et al. The START Study to evaluate the effectiveness of a combination intervention package to enhance antiretroviral therapy uptake and retention during TB treatment among TB/HIV patients in Lesotho: rationale and design of a mixed-methods, cluster-randomized trial. *Glob Health Action* 2016; **9**(1): 31543.

17. Story A, Garfein RS, Hayward A, et al. Monitoring Therapy Adherence of Tuberculosis Patients by using Video-Enabled Electronic Devices. *Emerg Infect Dis* 2016; **22**(3): 538-40.

18. Chuck C, Robinson E, Macaraig M, Alexander M, Burzynski J. Enhancing management of tuberculosis treatment with video directly observed therapy in New York City. *Int J Tuberc Lung Dis* 2016; **20**(5): 588-93.

19. Gassanov MA, Feldman LJ, Sebastian A, Kraguljac MJ, Rea E, Yaffe B. The Use of Videophone for Directly Observed Therapy for the Treatment of Tuberculosis. *Can J Public Health* 2013; **104**(3): e272.

20. Garfein RS, Collins K, Munoz F, et al. Feasibility of tuberculosis treatment monitoring by video directly observed therapy: a binational pilot study. *Int J Tuberc Lung Dis* 2015; **19**(9): 1057-64.

21. AiCure. LA County Department of Public Health: monitoring treatment to tuberculosis disease and latent tuberculosis infection. 2017. https://aicure.com/clinical_evidence/using-aicure-to-meausure-and-support-tuberculosis-treatment-a-pilot-study/ [accessed 2017 Oct 18].

22. Labovitz DL SL, Reyes GM, Virmani D, Hanina A. Using artificial intelligence to reduce the risk of nonadherence in patients on anticoagulation therapy. *Stroke* 2017; **48**(5): 1416-19.

23. Mirsaeidi M, Farshidpour M, Banks-Tripp D, Hashmi S, Kujoth C, Schraufnagel D. Video directly observed therapy for treatment of tuberculosis is patient-oriented and cost-effective. *Eur Respir J* 2015; **46**(3): 871-4.

24. Zuniga ML, Collins K, Munoz F, et al. A qualitative study exploring stakeholder perceptions of video directly observed therapy for monitoring tuberculosis treatment in the US-Mexico border region. *J Mob Technol Med* 2016; **5**(2): 12-23.

25. Hoffman JA, Cunningham JR, Suleh AJ, et al. Mobile direct observation treatment for tuberculosis patients: a technical feasibility pilot using mobile phones in Nairobi, Kenya. *Am J Prev Med* 2010; **39**(1): 78-80.

26. Sinkou H, Hurevich H, Rusovich V, et al. Video-observed treatment for tuberculosis patients in Belarus: findings from the first programmatic experience. *Eur Respir J* 2017; **49**(3).

27. Krueger K, Ruby D, Cooley P, et al. Videophone utilization as an alternative to directly observed therapy for tuberculosis. *Int J Tuberc Lung Dis* 2010; **14**(6): 779-81.

28. Wade VA, Karnon J, Eliott JA, Hiller JE. Home videophones improve direct observation in tuberculosis treatment: a mixed methods evaluation. *PLoS ONE* 2012; **7**(11): e50155.

29. Clinicaltrials.gov. Virtually Observed Treatment (VOT) for Tuberculosis Patients in Moldova, 2015.

30. ISRCTN registry. TB Reach 5: to compare the efficacy of video observed treatment (VOT) versus directly observed treatment (DOT) in supporting adherence in patients with active tuberculosis [ISRCTN26184967], 2014.

31. Checchi KD, Huybrechts KF, Avorn J, Kesselheim AS. Electronic medication packaging devices and medication adherence: a systematic review. *JAMA* 2014; **312**(12): 1237-47.

32. Liu X, Blaschke T, Thomas B, et al. Usability of a Medication Event Reminder Monitor System (MERM) by Providers and Patients to Improve Adherence in the Management of Tuberculosis. *Int J Environ Res Public Health* 2017; **14**(10).

33. Musiimenta A. Real time tuberculosis medication adherence intervention in rural southwestern Uganda. Uganda: Fogarty International Center, National Institutes of Health; 2017.

34. China Center for Disease Control and Prevention. A trial of an electronic pill box with reminders for patients taking treatment for tuberculosis (clinical trials registration), 2016.

35. Belknap R, Weis S, Brookens A, et al. Feasibility of an ingestible sensor-based system for monitoring adherence to tuberculosis therapy. *PLoS ONE* 2013; **8**(1): e53373.

36. Browne S, Moser K, Low J, et al. Wirelessly observed therapy is accurate and confirms more TB medication doses than directly observed therapy (abstract OA-137-12). In: Proceedings of the 48th Union World Conference on Lung Health [Internet]; 2017 October 11-14;

Guadalajara, Mexico. Guadalajara: The Union; 2017 [cited 2018 Mar 15]; p. S65. Available from: http://theunion2017.abstractmgmt.com/files2017/2371_Final UNION slides 10112017.pptx.

37. Au-Yeung KY, Moon GD, Robertson TL, et al. Early clinical experience with networked system for promoting patient self-management. *Am J Manag Care* 2011; **17**(7): e277-87.

38. Sharp RR. Ingestible Drug Adherence Monitors: Trending Toward a Surveillance Society? *Am J Bioeth* 2015; **15**(11): 1-2.

39. Belluck P. First digital pill approved to worries about biomedical "big brother". New York Times. 2017 November 13.

40. Au-Yeung KY, DiCarlo L. Cost comparison of wirelessly vs. directly observed therapy for adherence confirmation in anti-tuberculosis treatment. *Int J Tuberc Lung Dis* 2012; **16**(11): 1498-504.

41. Mkopi A, Range N, Lwilla F, et al. Adherence to tuberculosis therapy among patients receiving home-based directly observed treatment: evidence from the United Republic of Tanzania. *PLoS ONE* 2012; **7**(12): e51828.

42. Mkopi A, Range N, Lwilla F, et al. Validation of indirect tuberculosis treatment adherence measures in a resource-constrained setting. *Int J Tuberc Lung Dis* 2014; **18**(7): 804-9.

43. Nackers F, Huerga H, Espie E, et al. Adherence to self-administered tuberculosis treatment in a high HIV-prevalence setting: a cross-sectional survey in Homa Bay, Kenya. *PLoS One* 2012; **7**(3): e32140.

44. Meissner PE, Musoke P, Okwera A, Bunn JE, Coulter JB. The value of urine testing for verifying adherence to anti-tuberculosis chemotherapy in children and adults in Uganda. *Int J Tuberc Lung Dis* 2002; **6**(10): 903-8.

45. Whitfield R, Cope GF. Point-of-care test to monitor adherence to anti-tuberculous treatment. *Ann Clin Biochem* 2004; **41**(Pt 5): 411-3.

46. Soobratty MR, Whitfield R, Subramaniam K, et al. Point-of-care urine test for assessing adherence to isoniazid treatment for tuberculosis. *Eur Respir J* 2014; **43**(5): 1519-22.

47. Elizaga J, Friedland JS. Monitoring compliance with antituberculous treatment by detection of isoniazid in urine. *Lancet* 1997; **350**(9086): 1225-6.

48. Huan S, Chen R, Liu X, Ou X, Jiang S, Zhao Y. Operational feasibility of medication monitors in monitoring treatment adherence among TB patients. *Chin J Antituberculosis* 2012; **34**: 419-24.

49. Ruslami R, Aarnoutse RE, Alisjahbana B, Van Der Ven AJAM, Van Crevel R. Implications of the global increase of diabetes for tuberculosis control and patient care. *Trop Med Int Health* 2010; **15**(11): 1289-99.

50. Zentner I, Schlecht HP, Khensouvann L, et al. Urine colorimetry to detect Low rifampin exposure during tuberculosis therapy: a proof-of-concept study. *BMC Inf Dis* 2016; **16**: 242.

51. Bangsberg DR, Hecht FM, Charlebois ED, Chesney M, Moss A. Comparing Objective Measures of Adherence to HIV Antiretroviral Therapy: Electronic Medication Monitors and Unannounced Pill Counts. *AIDS Behav* 2001; **5**(3): 275-81.

52. van den Boogaard J, Lyimo RA, Boeree MJ, Kibiki GS, Aarnoutse RE. Electronic monitoring of treatment adherence and validation of alternative adherence measures in tuberculosis patients: a pilot study. *Bull World Health Organ* 2011; **89**(9): 632-9.

53. Agot K, Taylor D, Corneli AL, et al. Accuracy of Self-Report and Pill-Count Measures of Adherence in the FEM-PrEP Clinical Trial: Implications for Future HIV-Prevention Trials. *AIDS Behav* 2015; **19**(5): 743-51.

54. Kalichman SC, Amaral CM, Cherry C, et al. Monitoring medication adherence by unannounced pill counts conducted by telephone: reliability and criterion-related validity. *HIV Clin Trials* 2008; **9**(5): 298-308.

55. Hirsch-Moverman Y, Burkot C, Saito S, et al. Reaching the end of the line: Operational issues with implementing phone-based unannounced pill counts in resource-limited settings. *PLoS One* 2017; **12**(10): e0185549.

56. Thomas A, Gopi PG, Santha T, et al. Predictors of relapse among pulmonary tuberculosis patients treated in a DOTS programme in South India. *Int J Tuberc Lung Dis* 2005; **9**(5): 556-61.

57. Menzies D, Benedetti A, Paydar A, et al. Effect of duration and intermittency of rifampin on tuberculosis treatment outcomes: a systematic review and meta-analysis. *PLoS Med* 2009; **6**(9): e1000146.

58. Ahmad Khan F, Minion J, Al-Motairi A, Benedetti A, Harries AD, Menzies D. An updated systematic review and meta-analysis on the treatment of active tuberculosis in patients with HIV infection. *Clin Infect Dis* 2012; **55**(8): 1154-63.

59. Nahid P, Dorman SE, Alipanah N, et al. Official American Thoracic Society/Centers for Disease Control and Prevention/Infectious Diseases Society of America Clinical Practice Guidelines: Treatment of Drug-Susceptible Tuberculosis. *Clin Infect Dis* 2016; **63**(7): e147-e95.

60. World Health Organization (WHO). Definitions and reporting framework for tuberculosis -- 2013 revision (updated December 2014). Geneva: WHO, 2014.

61. U.S. Food and Drug Administration Center for Drug Evaluation and Research. Guidance for Industry. Pulmonary Tuberculosis: Developing Drugs for Treatment (Draft Guidance). Silver Spring, MD: Food and Drug Administration, 2013.

62. Narayanan S, Swaminathan S, Supply P, et al. Impact of HIV infection on the recurrence of tuberculosis in South India. *J Infect Dis* 2010; **201**(5): 691-703.

63. Charalambous S, Grant AD, Moloi V, et al. Contribution of reinfection to recurrent tuberculosis in South African gold miners. *Int J Tuberc Lung Dis* 2008; **12**(8): 942-8.

64. Nunn AJ, Phillips PP, Mitchison DA. Timing of relapse in short-course chemotherapy trials for tuberculosis. *Int J Tuberc Lung Dis* 2010; **14**(2): 241-2.
